# *In Vitro* Antibacterial Activity of Silver Nanoparticles Conjugated with Amikacin and Combined with Hyperthermia against Drug-Resistant and Biofilm-Producing Strains

**DOI:** 10.1128/spectrum.00280-23

**Published:** 2023-04-20

**Authors:** Marta Palau, Estela Muñoz, Muriel F. Gusta, Nieves Larrosa, Xavier Gomis, Joan Gilabert, Benito Almirante, Victor Puntes, Robert Texidó, Joan Gavaldà

**Affiliations:** a Antibiotic Resistance Laboratory, Vall d’Hebron Research Institute (VHIR), Infectious Diseases Department, Vall d’Hebron University Hospital, Barcelona, Spain; b Spanish Network for Research in Infectious Diseases (REIPI RD19/0016), Instituto de Salud Carlos III, Madrid, Spain; c CIBERINFEC, ISCIII-CIBER de Enfermedades Infecciosas, Instituto de Salud Carlos III, Madrid, Spain; d Institut Català de Nanociència i Nanotecnologia (ICN2), CSIC, The Barcelona Institute of Science and Technology (BIST), Campus UAB, Bellaterra, Barcelona, Spain; e Vall d’Hebron University Hospital, Barcelona, Spain; f CIBER en Bioingeniería, Biomateriales y Nanomedicina, CIBER-BBN, Madrid, Spain; g Microbiology Department, Vall d’Hebron University Hospital, Barcelona, Spain; h Tractivus SL, Barcelona, Spain; i Institució Catalana de Recerca i Estudis Avançats (ICREA), Barcelona, Spain; j Grup d’Enginyeria de Materials (GEMAT), Universitat Ramón Llull, Barcelona, Spain; JMI Laboratories

**Keywords:** antibacterial activity, MDR/XDR, amikacin, biofilms, hyperthermia, silver nanoparticles

## Abstract

In view of the current increase and spread of antimicrobial resistance (AMR), there is an urgent need to find new strategies to combat it. This study had two aims. First, we synthesized highly monodispersed silver nanoparticles (AgNPs) of approximately 17 nm, and we functionalized them with mercaptopoly(ethylene glycol) carboxylic acid (mPEG-COOH) and amikacin (AK). Second, we evaluated the antibacterial activity of this treatment (AgNPs_mPEG_AK) alone and in combination with hyperthermia against planktonic and biofilm-growing strains. AgNPs, AgNPs_mPEG, and AgNPs_mPEG_AK were characterized using a suite of spectroscopy and microscopy methods. Susceptibility to these treatments and AK was determined after 24 h and over time against 12 clinical multidrug-resistant (MDR)/extensively drug-resistant (XDR) isolates of Acinetobacter baumannii, Escherichia coli, Klebsiella pneumoniae, and Pseudomonas aeruginosa. The efficacy of the treatments alone and in combination with hyperthermia (1, 2, and 3 pulses at 41°C to 42°C for 15 min) was tested against the same planktonic strains using quantitative culture and against one P. aeruginosa strain growing on silicone disks using confocal laser scanning microscopy. The susceptibility studies showed that AgNPs_mPEG_AK was 10-fold more effective than AK alone, and bactericidal efficacy after 4, 8, 24, or 48 h was observed against 100% of the tested strains. The combination of AgNPs_mPEG_AK and hyperthermia eradicated 75% of the planktonic strains and exhibited significant reductions in biofilm formation by P. aeruginosa in comparison with the other treatments tested, except for AgNPs_mPEG_AK without hyperthermia. In conclusion, the combination of AgNPs_mPEG_AK and hyperthermia may be a promising therapy against MDR/XDR and biofilm-producing strains.

**IMPORTANCE** Antimicrobial resistance (AMR) is one of the greatest public health challenges, accounting for 1.27 million deaths worldwide in 2019. Biofilms, a complex microbial community, directly contribute to increased AMR. Therefore, new strategies are urgently required to combat infections caused by AMR and biofilm-producing strains. Silver nanoparticles (AgNPs) exhibit antimicrobial activity and can be functionalized with antibiotics. Although AgNPs are very promising, their effectiveness in complex biological environments still falls below the concentrations at which AgNPs are stable in terms of aggregation. Thus, improving the antibacterial effectiveness of AgNPs by functionalizing them with antibiotics may be a significant change to consolidate AgNPs as an alternative to antibiotics. It has been reported that hyperthermia has a large effect on the growth of planktonic and biofilm-producing strains. Therefore, we propose a new strategy based on AgNPs functionalized with amikacin and combined with hyperthermia (41°C to 42°C) to treat AMR and biofilm-related infections.

## INTRODUCTION

Antibiotics have transformed medicine and have become essential since their discovery, saving millions of lives every year (1, 2). However, the overuse and irrational use of antibiotics, coupled with the lack of development of new antibiotics, have led to the evolution of antibiotic-resistant microorganisms (2, 3). Antibiotic resistance is considered one of the greatest challenges worldwide in the health care field, affecting individuals of all ages (4). In 2019, 1.27 million deaths were attributed to antimicrobial resistance (AMR), 77,600 of them in Central Europe (5). The World Health Organization (WHO) has presented a priority list of antibiotic-resistant bacterial pathogens to help prioritize research and development for new strategies to treat AMR (6). The ESKAPE pathogens, including Enterococcus faecium, Staphylococcus aureus, Klebsiella pneumoniae, Acinetobacter baumannii, Pseudomonas aeruginosa, and Enterobacter spp., were classified as being critical and of high priority (6, 7). These pathogens are responsible for most of the nosocomial infections affecting ill and immunocompromised patients and have an increased potential to resist the bactericidal effect of antibiotics (7, 8). Furthermore, these pathogens are able to grow as a community of microorganisms enclosed by a self-produced extracellular polymeric matrix that confers great robustness (9, 10). This aggregate is referred to as a biofilm and can attach to a wide variety of the biotic and abiotic surfaces of medical devices and installations (11). Biofilms directly contribute to increased AMR, showing reduced susceptibility to antimicrobial agents in comparison with their planktonic counterparts (9, 10). Specifically, biofilms are 10- to 1,000-fold more resistant to antimicrobial agents than planktonic cells (9). Biofilm-associated infections have been estimated by the National Institutes of Health to represent approximately 80% of the infections in the body (12). Several pathogens are involved in chronic biofilm-associated infections, such as P. aeruginosa in cystic fibrosis pneumonia and Escherichia coli in recurrent urinary tract infections (12). Such persistent infections dramatically increase the morbidity and mortality of patients and represent a considerable economic burden for the health system (13). Therefore, the development of new strategies with novel mechanisms of action to withstand AMR and biofilm-producing strains causing infections is urgently required (14).

Today, scientific advances offer a promising platform for overcoming this problem by combining biological methods and nanotechnology tools (15). Nanomaterials are currently designed to facilitate the transport of therapeutic agents through biological barriers or to improve their targeting by promoting certain molecular interactions or detecting molecular changes in a more sensitive manner (15). Their surfaces can be functionalized with several types of molecules, allowing them to be used as precision carriers (15). Thus, functionalization may facilitate the penetration of molecules into biofilms and, consequently, reduce biofilm formation (16). In this regard, metal nanoparticles (NPs) are among the most promising nanomaterials owing to their outstanding chemical and physical properties, which offer a wide range of applications in biomedical engineering, biosensing, pharmacy, and nanomedicine (17). They can be used to convey antimicrobials, they can be involved in the delivery of drugs, or, for silver nanoparticles (AgNPs), they can act as antimicrobials themselves (18, 19). AgNPs have been reported to exhibit high antimicrobial efficacy against bacteria, viruses, and fungi (18, 19).

Although the broad-spectrum antimicrobial properties of AgNPs have been extensively reported, clear and definitive knowledge of the effects of AgNPs on microorganisms is still lacking (17, 20–23). An increasing body of evidence indicates that the mechanism of action is based on three routes, which are often related to one another and can occur simultaneously (17, 20–23). The first is based on direct contact between AgNPs or Ag^+^ and the bacterial membrane, causing changes in permeability and structure and the rupture and consequent loss of the cytoplasmic content (17, 20–23). The second route encompasses the entrance of AgNPs and Ag^+^ into the cell, which can destabilize different biomolecules (proteins, lipids, and DNA) and ribosomes and interfere with some essential processes in the bacterial cell, such as protein synthesis, the functioning of the respiratory chain, the production of ATP, gene transcription, and DNA replication (17, 20–23). Through the third route, the corrosion and natural transformation of AgNPs into Ag^+^ when dispersed in biological media increase the production of reactive oxygen species (ROS) and the consequent oxidation of proteins, lipids, and DNA breaks (17, 20–23). Therefore, AgNPs have been suggested as a viable alternative for the treatment of infections, especially those caused by multidrug-resistant (MDR) and extensively drug-resistant (XDR) bacteria and biofilms (21, 24).

The use of heat as a therapeutic method has been repeatedly described in the medical literature over the past centuries, especially for use in cancer treatments (25). Hyperthermia is defined as an increase in temperature that provides therapeutic benefits without causing cellular death (25). This temperature increase ranges from 41°C to 50°C (25). Apart from the effect of hyperthermia on cancer therapies, it has been demonstrated to be effective against bacteria (26, 27). Furthermore, some studies have indicated that hyperthermia can lead to bacterial biofilm death (28). In this regard, metallic NPs such as AgNPs are known to be excellent local conductors of heat, which makes them effective for transmitting heat to adjacent tissues (29). Currently, the application of AgNPs without another bactericidal agent is not effective against biofilms. At high concentrations, AgNPs suffer from aggregation phenomena in complex biological media, which reduce their bactericidal efficacy (30). To solve this problem, in this study, AgNPs have been modified with amikacin (AK) to improve their effectiveness and combined with hyperthermia treatments to go a step further in biofilm removal. Therefore, we designed a multimodal strategy to combat antibiotic-resistant bacteria based on the application of AgNPs conjugated with mercaptopoly(ethylene glycol) carboxylic acid (mPEG-COOH) and AK, combined with hyperthermia.

This study had two aims. First, we synthesized and characterized highly monodispersed AgNPs and linked them with mPEG-COOH and AK. Second, we evaluated the *in vitro* susceptibility of a wide range of MDR and XDR clinical bacterial strains growing planktonically and in biofilms to AgNPs conjugated with mPEG and AK (AgNPs_mPEG_AK) alone and in combination with hyperthermia at 41°C to 42°C.

## RESULTS

### Characterization of the synthesized AgNPs.

**FIG 1:**
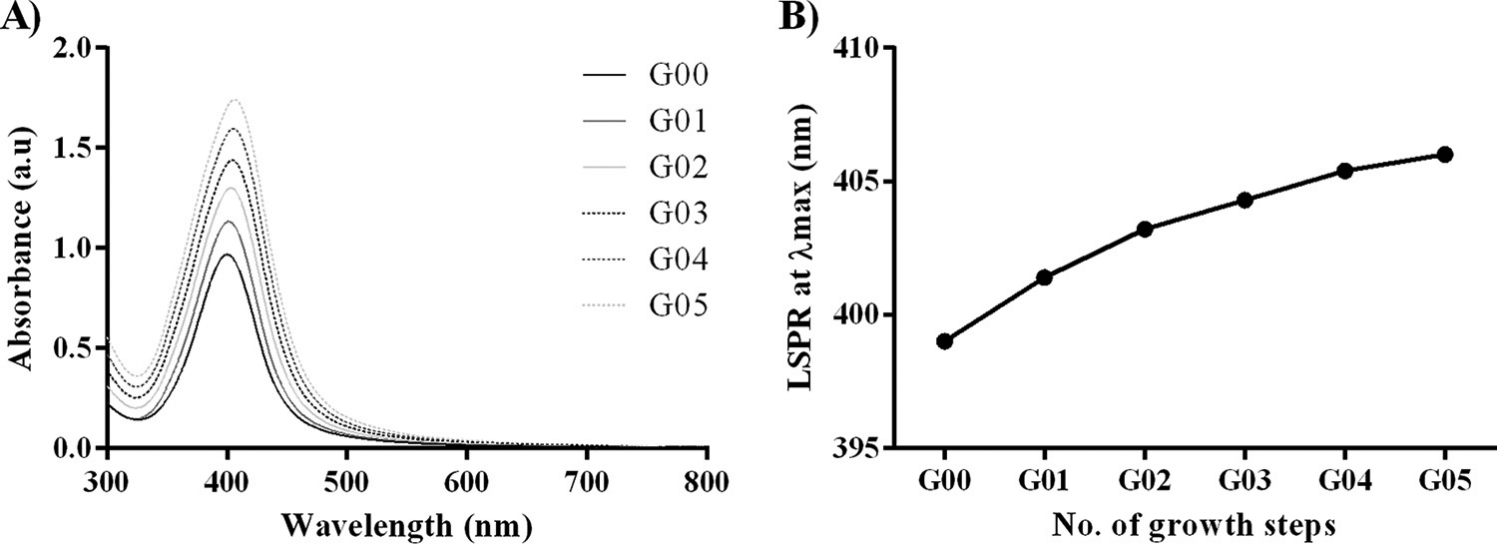
UV-Vis spectrum results for synthesized AgNPs using the seed growth technique. (A) Evolution of the UV-Vis spectra of the AgNPs during the different growth steps (G00 to G05). a.u, arbitrary units. (B) Localized surface plasmon resonance (LSPR) peak positions plotted as a function of the growth step (G00 to G05). G00, nucleation; G01, first growth step; G02, second growth step; G03, third growth step; G04, fourth growth step; G05, fifth growth step.

The UV-visible (UV-Vis) spectra of the AgNPs obtained from seeded growth synthesis are shown in Fig. 1. A redshift of the localized surface plasmon resonance (LSPR) peak of the AgNPs to longer wavelengths was observed at each growth step (G00 to G05), correlating with increases in the sizes of the NPs. Furthermore, the narrow absorption peaks confirmed the high monodispersity of AgNPs. As the maximum intensity depends linearly on the size of the AgNPs, the increasing maximum absorbances of the different growth steps indicate uniform growth. Therefore, the injected atoms of Ag were deposited onto the surface of seed particles, avoiding new nucleation and focusing the size distribution, achieving highly monodispersed AgNPs.

**FIG 2:**
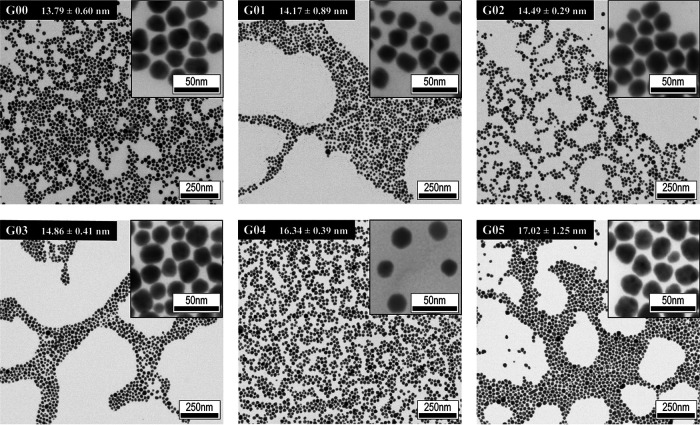
Transmission electron microscopy (TEM) images of the synthesized AgNPs in the nucleation and growth steps and sizes of the AgNPs at every growth step. G00, nucleation; G01, first growth step; G02, second growth step; G03, third growth step; G04, fourth growth step; G05, fifth growth step.

The morphology and size of the AgNPs were confirmed using transmission electron microscopy (TEM) (Fig. 2). The TEM images showed that the synthesized AgNPs were highly uniform in size and shape. The size of the AgNPs progressively increased at each growth step, from 13.79 ± 0.60 nm at the nucleation step (G00) to 17.02 ± 1.25 nm at the last growth step (G05).

### Characterization of AgNPs_mPEG and AgNPs_mPEG_AK.

The UV-Vis spectra and dynamic light scattering (DLS) results are presented in Table 1. After conjugating AgNPs (G05) with mPEG-COOH and AK, a redshift of the LSPR peak toward longer wavelengths was observed. This can be ascribed to the change in the refractive index in the vicinity of the particles, which is consistent with the binding of mPEG-COOH through a thiol bond to the Ag surface. The modification of AgNPs with mPEG and AK leads to a change in particle size. Accordingly, the increases in the sizes of AgNPs_mPEG (DLS, 33.3 ± 8.2 nm; nanoparticle tracking analysis [NTA], 59.2 ± 24.8 nm) and AgNPs_mPEG_AK (DLS, 39.8 ± 10.3 nm; NTA, 72.3 ± 38.5 nm) compared to AgNPs (DLS, 18.9 ± 2.2 nm; NTA, 48.2 ± 15.1 nm) were also verified by DLS and NTA. Moreover, regarding the zeta potential (ZP) measurements, since more positive values were obtained after conjugation (AgNPs_mPEG, −30.5 ± 5.6 mV; AgNPs_mPEG_AK, −21.7 ± 3.7 mV), the functionalization of mPEG and AK on the surface of AgNPs (−46.3 ± 6.3 mV) was confirmed. All measurements were performed under the same pH and ionic strength conditions.

**TABLE 1:** Results for the optical properties, sizes, and surface charges of the AgNPs, AgNPs_mPEG, and AgNPs_mPEG_AK^*a*^

Treatment	LSPR peak (nm)	Mean size (nm) by DLS (I) ± SD	Mean size (nm) by NTA ± SD	Mean [NPs] (particles/mL) ± SD	Mean zeta potential (mV) ± SD	Mean [AK] (mg/L) ± SD
AgNPs	406.7	18.9 ± 2.2	48.2 ± 15.1	1.19 × 10^9^ ± 2.66 × 10^7^	−46.3 ± 6.3	0
AgNPs_mPEG	407.8	33.3 ± 8.2	59.2 ± 24.8	1.54 × 10^9^ ± 9.05 × 10^7^	−30.5 ± 5.6	0
AgNPs_mPEG_AK	408.3	39.8 ± 10.3	72.3 ± 38.5	1.47 × 10^9^ ± 9.19 × 10^7^	−21.7 ± 3.7	1.01 ± 0.69

a mPEG, mercaptopoly(ethylene glycol) carboxylic acid; AK, amikacin; AgNPs, silver nanoparticles; LSPR, localized surface plasmon resonance; DLS, dynamic light scattering; I, intensity; NTA, nanoparticle tracking analysis; [NPs], concentration of nano particles; [AK], concentration of amikacin.

The concentrations of particles in the three instances (AgNPs, AgNPs_mPEG, and AgNPs_mPEG_AK) were similar: 1.19 × 10^9^ ± 2.66 × 10^7^ particles/mL for AgNPs, 1.54 × 10^9^ ± 9.05 × 10^7^ particles/mL for AgNPs_mPEG, and 1.47 × 10^9^ ± 9.19 × 10^7^ particles/mL for AgNPs_mPEG_AK.

Regarding AgNPs_mPEG_AK, the concentration of AK that interacts with AgNPs_mPEG, determined using the quantitative microsphere system (QMS) amikacin assay, was 1.01 ± 0.69 mg/L.

### Planktonic susceptibility studies.

**(i) Efficacy of AgNPs_mPEG_AK determined by microdilution technique.** The results of the determination of MICs are shown in Table 2.

**TABLE 2:** *In vitro* susceptibility of A. baumannii, E. coli, K. pneumoniae, and P. aeruginosa strains to AgNPs_mPEG, AgNPs_mPEG_AK, AgNPs_mPEG+AK, and AK^*a*^

Strain	MIC (mg/L)			
	AgNPs_mPEG	AgNPs_mPEG_AK	AgNPs_mPEG+AK	AK
A. baumannii				
Ab4	>12.5	0.5	4	4
Ab60	>12.5	0.5	2	4
Ab4249	>12.5	5	32	64
E. coli				
Ec1	6.25	0.06	1	4
Ec6	6.25	0.125	2	8
Ec13	3.125	0.125	2	8
K. pneumoniae				
Kp3	6.25	0.5	32	64
Kp6	>12.5	0.25	2	4
Kp16	>12.5	0.125	0.5	0.5
P. aeruginosa				
Pa3	3.125	0.25	32	128
Pa46	3.125	0.25	16	64
Pa1016	6.25	0.25	2	4

a MIC, minimum inhibitory concentration; AgNPs, silver nanoparticles; mPEG, mercaptopoly(ethyleneglycol) carboxylic acid; AK, amikacin.

Notably, for 12 out of all 12 strains (100%), treatment with AgNPs_mPEG_AK showed the highest efficacy in comparison with the other treatments tested. Moreover, AgNPs_mPEG_AK was at least 10-fold more effective than AK alone. Furthermore, this behavior also occurred in AK-resistant strains, and even 100% of the resistant strains (Ab4249, Kp3, Pa3, and Pa46) became susceptible to AK by treatment with AgNPs_mPEG_AK.

The treatment with a mixture of AgNPs_mPEG+AK (AK nonconjugated) was ≥2-fold more effective than AK alone in all of the tested strains, except for Ab4 and Kp16.

### (ii) Efficacy of AgNPs_mPEG, AgNPs_mPEG_AK, AgNPs_mPEG plus AK, and AK alone over time.

**FIG 3:**
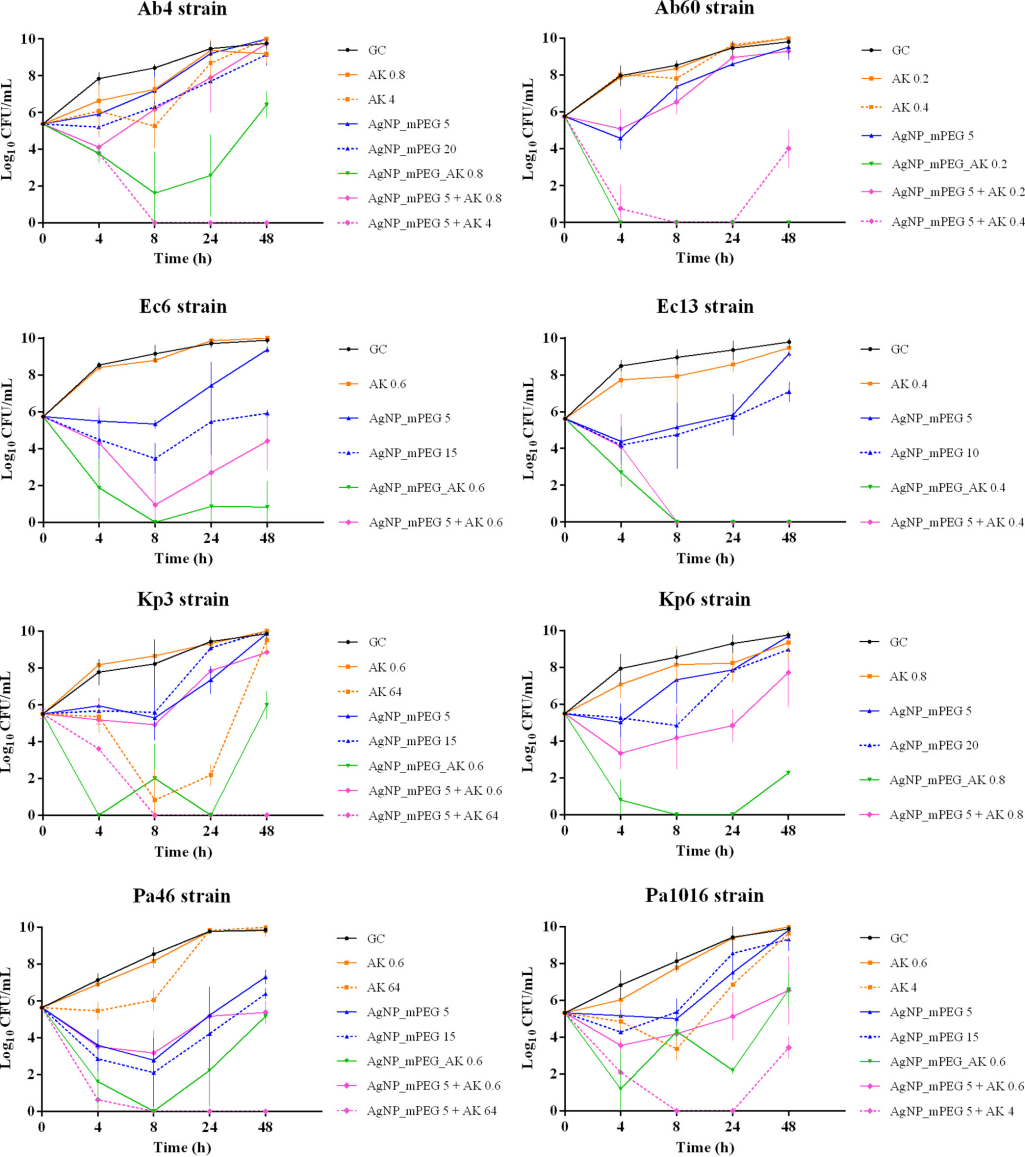
Results for the susceptibility of A. baumannii (Ab4 and Ab60), E. coli (Ec6 and Ec13), K. pneumoniae (Kp3 and Kp6), and P. aeruginosa (Pa46 and Pa1016) to silver nanoparticles (AgNPs) conjugated with mercaptopoly(ethylene glycol) carboxylic acid (mPEG) (AgNPs_mPEG), AgNPs conjugated with mPEG and amikacin (AK) (AgNPs_mPEG_AK), AgNPs_mPEG+AK, and AK at different concentrations over time (4, 8, 24, and 48 h). The concentrations of the treatments are expressed in milligrams per liter. GC, growth control.

Figure 3 shows the efficacy of AgNPs_mPEG_AK and AgNPs_mPEG+AK over time against different strains of A. baumannii, E. coli, K. pneumoniae, and P. aeruginosa.

Treatment with AgNPs_mPEG_AK revealed a bactericidal effect on Ab60, Ec6, and Kp6 at 4, 8, 24, and 48 h. A bactericidal effect on Kp3 and Pa46 was also observed at 4, 8, and 24 h posttreatment. For Ec13 and Pa1016, the bactericidal effects of AgNPs_mPEG_AK were observed at 8, 24, and 48 h and at 4 and 24 h, respectively. Finally, a bactericidal effect on Ab4 was observed only at 8 h posttreatment.

Comparing AgNPs_mPEG+AK to the treatments alone at the same concentrations, we observed a synergistic effect at 4, 8, 24, and 48 h posttreatment against Ab4, Ab60, Pa46, and Pa1016. This effect was also observed for Ec6 and Ec13 at 8, 24, and 48 h posttreatment. Finally, a synergistic effect was observed with this treatment at 24 and 48 h and 8 and 24 h posttreatment against Kp3 and Kp6, respectively.

Furthermore, AgNPs_mPEG_AK caused a greater reduction in bacterial growth at 4, 8, 24, or 48 h posttreatment than with the combination of AgNPs_mPEG+AK (AK nonconjugated) in 100% of the tested strains, highlighting the powerful effect of AgNPs_mPEG_AK (AK conjugated).

### (iii) Efficacy of AgNPs_mPEG_AK and hyperthermia.

**FIG 4:**
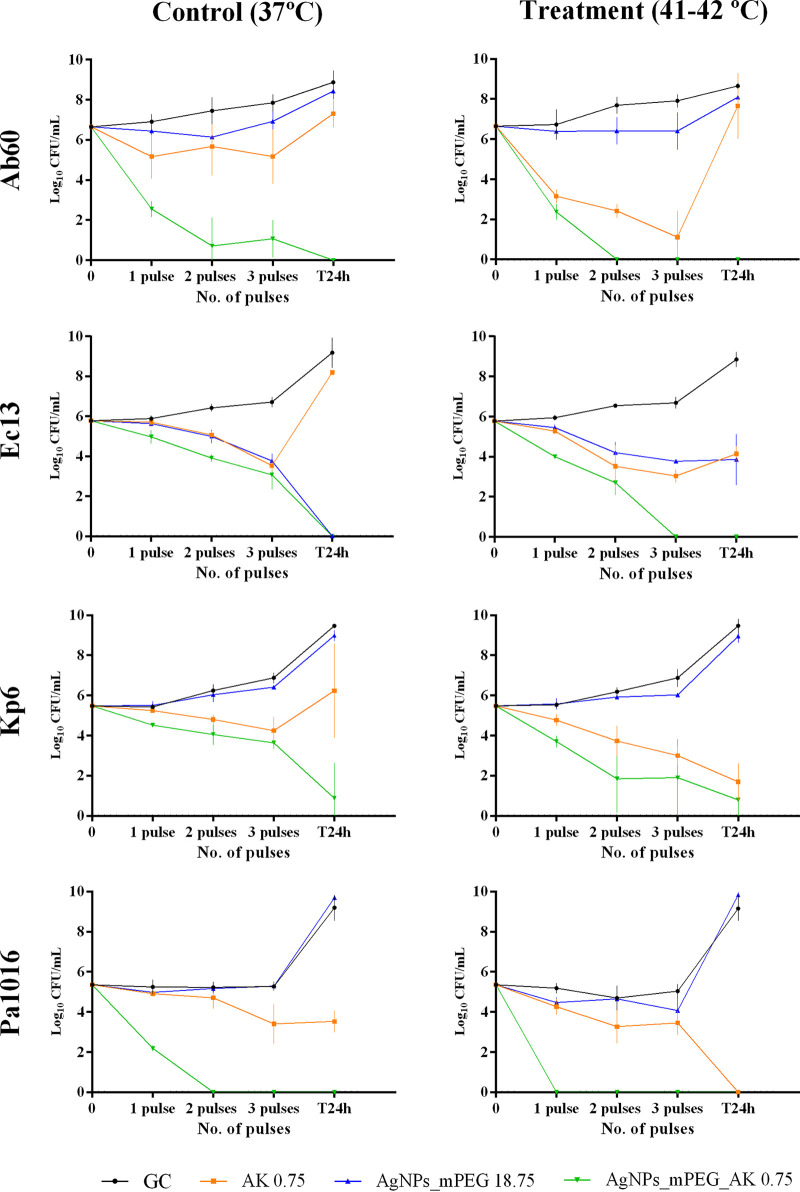
Results for the susceptibility of A. baumannii (Ab60), E. coli (Ec13), K. pneumoniae (Kp6), and P. aeruginosa (Pa1016) to silver nanoparticles (AgNPs) conjugated with mercaptopoly(ethylene glycol) carboxylic acid (mPEG) (AgNPs_mPEG) (18.75 mg/L), AgNPs conjugated with mPEG and amikacin (AK) (AgNPs_mPEG_AK) (0.75 mg/L of AK and 18.75 mg/L of Ag^+^), and AK (0.75 mg/L) alone (37°C) or in combination with hyperthermia (1, 2, and 3 pulses at 41°C to 42°C for 15 min each). GC, growth control.

The efficacy of the combination of AgNPs_mPEG_AK and hyperthermia is summarized in Fig. 4.

Of the tested strains, 75% (Ab60, Ec13, and Pa1016) were totally eradicated by the combination of AgNPs_mPEG_AK and hyperthermia. In all instances, the application of AgNPs_mPEG_AK combined with hyperthermia (41°C to 42°C) demonstrated greater efficacy in killing bacteria, as fewer pulses at 41°C to 42°C were needed than with the treatment without hyperthermia (37°C). Specifically, AgNPs_mPEG_AK plus 1, 2, or 3 pulses at 41°C to 42°C for 15 min for each pulse were needed to eradicate the Pa1016, Ab60, or Ec13 strain, respectively. Finally, Kp6 was the least susceptible to the effect of the combination of AgNPs_mPEG_AK plus 3 pulses at 41°C to 42°C for 15 min each, although a reduction of 8.68 log_10_ CFU/mL was achieved after 24 h in comparison with the growth control (GC) group plus hyperthermia (41°C to 42°C).

### Biofilm susceptibility studies: efficacy of AgNPs_mPEG_AK and hyperthermia determined by CLSM.

**FIG 5:**
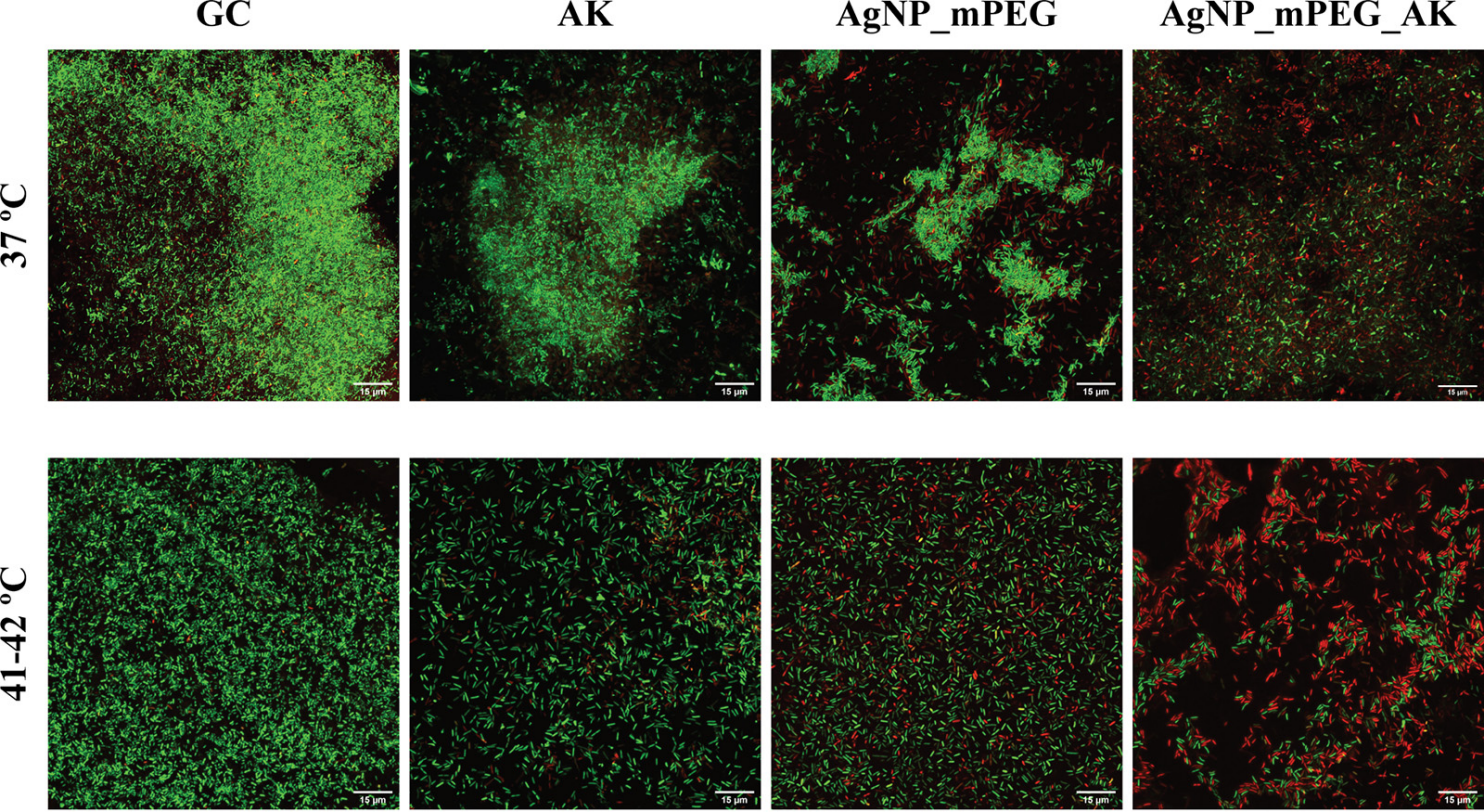
*In vitro* efficacy of silver nanoparticles (AgNPs) conjugated with mercaptopoly(ethylene glycol) carboxylic acid (mPEG) (AgNPs_mPEG) (75 mg/L), AgNPs conjugated with mPEG and amikacin (AK) (AgNPs_mPEG_AK) (3 mg/L of AK and 75 mg/L of Ag^+^), and AK (3 mg/L) alone (37°C) or in combination with hyperthermia (3 pulses at 41°C to 42°C for 15 min each) against a biofilm-producing strain of P. aeruginosa (Pa1016). Images were captured using confocal laser scanning microscopy (CLSM) and a Live/Dead staining kit (visualized at a ×60 magnification). Red fluorescence, dead cells; green fluorescence, live cells; GC, growth control.

**FIG 6:**
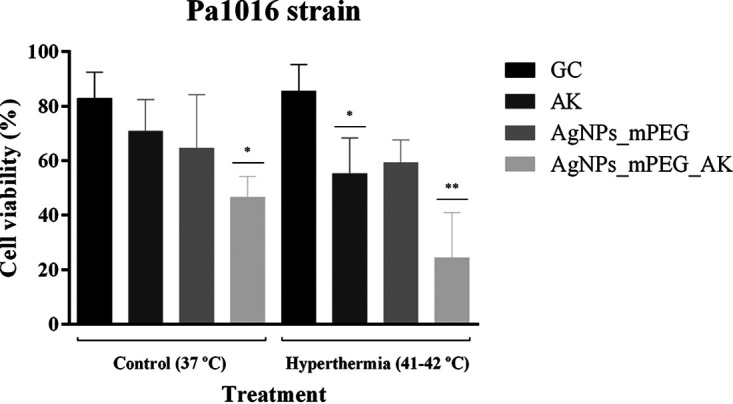
Efficacy of silver nanoparticles (AgNPs) conjugated with mercaptopoly(ethylene glycol) carboxylic acid (mPEG) (AgNPs_mPEG) (75 mg/L), AgNPs conjugated with mPEG and amikacin (AK) (AgNPs_mPEG_AK) (3 mg/L of AK and 75 mg/L of Ag^+^), and AK (3 mg/L) alone (37°C) or in combination with hyperthermia (3 pulses at 41°C to 42°C for 15 min each) against one biofilm-producing strain of P. aeruginosa (Pa1016). Cell viability was quantified from the images obtained by confocal laser scanning microscopy (CLSM) using a Live/Dead staining kit and is expressed as the mean percentage ± SD. *, *P* ≤ 0.05 versus GC (37°C) and GC plus hyperthermia; **, *P* ≤ 0.05 versus GC (37°C), GC plus hyperthermia, AK (37°C), AK plus hyperthermia, AgNPs_mPEG (37°C), and AgNPs_mPEG plus hyperthermia. GC, growth control.

The efficacy of AgNPs_mPEG, AgNPs_mPEG_AK, and AK combined with hyperthermia against a biofilm-producing strain of P. aeruginosa (Pa1016) is shown in Fig. 5 and 6.

Confocal laser scanning microscopy (CLSM) images revealed the potential bactericidal effect of the combination of AgNPs_mPEG_AK and hyperthermia, as more cells were killed (shown in red) than with the treatments with and without hyperthermia. Specifically, the cell viability counts from the CLSM images revealed that treatment with AgNPs_mPEG_AK plus 3 pulses at 41°C to 42°C for 15 min for each pulse was the only treatment that caused a significant reduction (24.42%) in cell viability in comparison with all of the other tested treatments, except for AgNPs_mPEG_AK without hyperthermia (Fig. 6). Note that AgNPs_mPEG_AK without hyperthermia (46.62%) showed a significant reduction in cell viability but only against its growth control.

## DISCUSSION

The aims of this study were as follows. First, we synthesized and characterized reproducible, highly monodispersed AgNPs, and next, we conjugated them with mPEG-COOH and AK. Second, we determined the *in vitro* antibacterial efficacy of AgNPs_mPEG_AK alone and in combination with hyperthermia (41°C to 42°C) against MDR/XDR Gram-negative bacteria growing as planktonic cells and in biofilms on the surface of silicone disks.

Although sodium citrate (SC) is a weak reducer, it can lead to the formation of larger and polydispersed AgNPs (20). The combined use of SC and tannic acid (TA) (strong reducer) has allowed us to obtain highly reproducible and monodispersed AgNPs in size and shape, as previously reported and observed using TEM.

The control of the shape, size, and surface charge of AgNPs is essential as they directly influence the bactericidal properties of AgNPs (20). We have controlled the size and shape of the synthesized AgNPs by combining traces of TA and SC to obtain spherical AgNPs with a size of approximately 17 nm. As described previously by Acharya et al. (31), although both spherical and rod-shaped AgNPs were good candidates as antimicrobial agents, spherical AgNPs revealed higher killing kinetics against a K. pneumoniae strain than with rod-shaped AgNPs (31). The antimicrobial activity of AgNPs relies on their ability to release Ag^+^ to the bacteria, as determined by the corrosion rate, which depends on the morphology (size and shape) of the NPs (20, 32). Accordingly, it has also been reported that smaller AgNPs have more powerful bactericidal activity as the area of surface contact of AgNPs with microorganisms is increased in relation to larger AgNPs (20, 23). This phenomenon was studied previously by Raza et al. (33), who synthesized spherical AgNPs of different sizes (15 to 50, 25 to 70, 30 to 80, 30 to 200, and 150 nm). They found that the smallest AgNPs (15 to 50 nm) displayed the strongest bactericidal effect on P. aeruginosa and E. coli strains in comparison with the larger AgNPs and antibiotics (33). Note that SC, in this instance, is used as a reducing and NP-stabilizing agent, which can simply be replaced when surface functionalization is required. Similar results were obtained previously by Bastús et al. (34), who deeply studied the role of TA and SC in the synthesis of AgNPs (34). They concluded that the use of both reductants allowed the accurate control of the size and shape of the AgNPs (35).

Moreover, the functionalization of AgNPs with mPEG-COOH and AK has been studied and confirmed by different characterization techniques, which revealed the redshift of the SPR peak and the change of the surface charge of AgNPs from values of −46.3 ± 6.3 mV (AgNPs) to values of −21.7 ± 3.7 mV (AgNPs_mPEG_AK). The use of SC for the synthesis and electrostatic stability of AgNPs allows us to easily functionalize the surface of AgNPs as citrate ions can be simply exchanged with other molecules with a higher affinity for the NP surface, such as mPEG-COOH (35). The pegylation of AgNPs has been previously studied in depth to increase stability and avoid aggregation between particles as it provides steric repulsion to the NPs, which is critical for preserving their colloidal stability in highly saline media such as culture broth (36). It can also reduce the opsonization of the NPs *in vivo*; therefore, the uptake of AgNPs by the reticuloendothelial system leads to an increase in their time of circulation (37). These are the reasons why AgNPs were first conjugated with mPEG-COOH in this study. Moreover, the presence of the thiol group in mPEG-COOH can facilitate the introduction of other molecules such as AK to enhance biocompatibility, reduce toxicity, serve as a carrier, and increase the time of circulation of the particles in the body (36).

As has been described in the literature, several antibiotics with different mechanisms of action are more effective against bacterial species when combined with AgNPs (38–41). We tested the antimicrobial susceptibility of the combination of AgNPs_mPEG and AK (AgNPs_mPEG+AK). Therefore, we were able to decrease the MIC values of AK by at least 50% in 10 out of 12 of the studied strains. Although a decrease in the MIC values for the AK-resistant strains was observed using this combination, the strains were still resistant to AK. Moreover, regarding efficacy studies over time, we observed a synergistic effect of AgNPs_mPEG+AK versus the treatments alone at 4, 8, 24, or 48 h posttreatment against all of the tested strains. Therefore, the antibacterial synergy of this combination may be explained by a combination of the mechanisms of action of AgNPs and AK, affecting bacterial growth (42). This synergistic effect was also observed previously by Alotaibi et al. (43), who combined AgNPs with antibiotics, including kanamycin, colistin, rifampicin, and vancomycin, against wild-type and AMR K. pneumoniae strains (43).

It has also been reported that AgNPs conjugated with some antibiotics can enhance the release of Ag^+^, which will cause a greater increase in the antimicrobial activity of the conjugate than with AgNPs or antibiotics alone (44). In our study, we also conjugated AgNPs with AK (AgNPs_mPEG_AK). Although AK is not the aminoglycoside of choice in clinical practice, it was chosen because it is one of the few antibiotics effective against MDR and XDR microorganisms that cause infections in patients. Moreover, the low concentration of AK in AgNPs (1 mg/L) and its expected local administration at the site of infection may reduce the risk of antibiotic nephrotoxicity and avoid the problem of its short half-life. In this manner, the MIC values of AgNPs_mPEG_AK were more than 10-fold lower than those of AK alone for all tested strains. Moreover, the studied strains that were resistant to AK (Ab4249, Kp3, Pa3, and Pa46) became susceptible to it when they were treated with AgNPs_mPEG_AK. These results correlate with those of Kaur and Kumar (44), who tested the bactericidal effect of AgNPs conjugated with AK on S. aureus and E. coli and observed an enhanced effect of the complex in comparison with the antibiotic alone (44). The results of our study also correlate with those of a previous study by Camargo et al. (45), who studied the antibacterial activity of AgNPs functionalized with AK and obtained an MIC value of <0.5 μg/mL against MDR A. baumannii strains, which was similar to our values. However, in our study, we also obtained promising results against other MDR/XDR clinical strains.

Moreover, in our study, we also observed a bactericidal effect using AgNPs_mPEG_AK after 4, 8, 24, or 48 h on all of the tested strains in comparison with the treatments alone. Furthermore, AgNPs_mPEG_AK caused a greater reduction in bacterial growth at 4, 8, 24, or 48 h posttreatment than with the combination of AgNPs_mPEG+AK (AK nonconjugated) in 100% of the tested strains, highlighting the powerful effect of AgNPs_mPEG_AK (AK conjugated). Therefore, considering the susceptibility results obtained, it appears that conjugating the antibiotic enhances the antimicrobial activity more than simply combining it as an adjuvant. Although regrowth has been observed in some tested strains using AgNPs_mPEG_AK, we think that this phenomenon could be solved by administering the treatment for shorter times. It would also be interesting to study the behavior of AgNPs_mPEG_AK and its administration in animal models in the future.

Metal NPs are distinguished by their excellent thermal conductivity, which allows the efficient transmission of heat to their surroundings and may potentiate their antimicrobial effect (29). Our results showed that the use of AgNPs_mPEG_AK combined with hyperthermia (41°C to 42°C) killed 75% of the tested strains. Notably, fewer pulses were required to eradicate the studied strains than with the treatments without hyperthermia (maintained at 37°C). The enhancement of the antibacterial effect of Ag^+^ by heat has also been studied by D’Agostino et al. (46). They studied the effect of Ag^+^ from Ag nanoplates grafted onto amino-functionalized bulk glass surfaces on strains of E. coli and S. aureus (46). They found that the combination of Ag^+^ and heat had higher antibacterial activity against the above-mentioned strains than with nonheated ones (46). However, they obtained a maximum reduction of only 3.6 log_10_ CFU/mL with the strategy against one strain of E. coli and one strain of S. aureus, and both strains were from the American Type Culture Collection (ATCC) (46). Conversely, in our study, we obtained a reduction of at least 4 to 5 log_10_ CFU/mL in comparison with the growth control against four different strains, all of which were obtained from patients at the Vall d’Hebron University Hospital (VHUH).

Furthermore, in our study, we explored the antibiofilm activity of the synthesized AgNPs against one XDR biofilm-producing strain of P. aeruginosa. AgNPs have been reported to have antibiofilm activity against Gram-negative and Gram-positive bacteria (22), although it has been observed that Gram-negative bacteria are more susceptible to AgNPs because of their outer membrane: the lipopolysaccharides and peptidoglycan from the membrane provide a negative charge that facilitates the electrostatic interactions between the AgNPs or Ag^+^ and the membrane of bacteria (22, 47).

Some studies suggest that AgNPs can be responsible for biofilm destruction by recognizing peptidoglycan and binding to the exopolysaccharide matrix, causing physical and morphological damage (48–50). Upon NP adhesion, Ag^+^ is released, which causes oxidative stress through ROS production and DNA damage, thereby disrupting the biofilm structure (48). Specifically, similar to our results, Singh et al. (49) showed that spherical AgNPs of 15 to 30 nm reduced biofilm formation by P. aeruginosa and E. coli (49). Similarly, another study, by Zhang et al. (50), observed inhibition of biofilm formation by P. aeruginosa using AgNPs (50).

Regarding the effect of AgNPs_mPEG_AK and hyperthermia on a biofilm-producing strain of P. aeruginosa, this was the only treatment that achieved a significant reduction in cell viability (24.42%) in comparison with the other tested treatments (GC, 82.87%; AK, 70.76%; AgNPs_mPEG, 64.61%; GC plus hyperthermia, 85.56%; AK plus hyperthermia, 55.25%; AgNPs_mPEG plus hyperthermia, 59.31%). Moreover, according to our results, it seems that the combination of AgNPs_mPEG and hyperthermia can cause biofilm disaggregation. The bimodal effect of hyperthermia and AgNPs, together with gold NPs (AuNPs), on biofilm-producing strains was also studied previously by Pallavicini et al. (51). They found that the use of monolayer near-infrared active silica-coated Au nanostars decorated with AgNPs had a synergistic antibacterial effect on S. aureus and E. coli strains growing in biofilms (51). The effect obtained was suggested to be based on the formation of pores in the cell wall, which disrupted the cell morphology of the studied strains (51). Although that study and ours have obtained promising results, our strategy was a simplification of that approach, as only one type of NP was needed to achieve the antibiofilm effect, whereas the study by Pallavicini et al. required a combination of both AgNPs and AuNPs. Moreover, as the hyperthermia results have been very promising, further studies will be focused on how to apply hyperthermia in animal models. Safety and efficacy studies of hyperthermia will be a challenge needed to get closer to therapeutic application.

In conclusion, the application of AgNPs conjugated with mPEG-COOH and AK and combined with hyperthermia appears to be a promising strategy to inhibit the growth of MDR/XDR bacteria as planktonic cells and biofilms. Furthermore, this combination allows opportunities for new therapies that provide greater antibacterial and antibiofilm efficacies since the use of this multimodal strategy might hinder the development of resistance to all three strategies.

## MATERIALS AND METHODS

### Strains and resistance mechanisms.

For planktonic susceptibility studies, 12 clinical MDR/XDR Gram-negative isolates were studied: 3 strains of A. baumannii (Ab4, Ab60, and Ab4249), 3 strains of E. coli (Ec1, Ec6, and Ec13), 3 strains of K. pneumoniae (Kp3, Kp6, and Kp16), and 3 strains of P. aeruginosa (Pa3, Pa46, and Pa1016). Sequence types, the main acquired β-lactam resistance mechanisms, and the results of antibiotic susceptibility testing (AST) of these Gram-negative strains are summarized in Table 3. The antimicrobial susceptibility of the strains was studied using the disk diffusion test (i2a, Montpellier, France) according to European Committee on Antimicrobial Susceptibility Testing (EUCAST) guidelines (52). In addition, susceptibility to antibiotics that are potentially active against some of these MDR/XDR isolates (ceftazidime, ceftazidime-avibactam, ceftolozane-tazobactam, piperacillin-tazobactam, cefiderocol, meropenem, meropenem-vaborbactam, imipenem-relebactam, amikacin, ciprofloxacin, trimethoprim-sulfamethoxazole, and colistin) was determined in duplicate by the microdilution technique using the Sensititre Gram-negative GN4F AST plate (Thermo Fisher Diagnostics SLU, Madrid, Spain). The discordance between results using previous techniques was confirmed using a gradient test (Etest; bioMérieux SA, Marcy l’Etoile, France). P. aeruginosa ATCC 27853, E. coli ATCC 25922, and K. pneumoniae ATCC 700603 and ATCC 2814 were used as quality control strains to determine the MICs.

**TABLE 3:** Characteristics of the Gram-negative isolates used in the study^*a*^

Strain	ST	3rd-generation cephalosporin and carbapenemase resistance mechanism(s)	MIC by microdilution technique (mg/L) and susceptibility									Disk diffusion zone diam (mm)		
			CTZ	CTV	CTT	PIT	MER	MEV	IMR	CIP	COL	FDC	AMI	TRS
Ab4	2	OXA-51	>32 ND	>8/4 ND	>8/4 ND	>64/4 ND	>16 R	>8/8 ND	>8/4 R	>4 R	1 S	20.5 R	22 S	6 R
Ab60	38	OXA-51	2 ND	>8/4 ND	>8/4 ND	>64/4 ND	2 S	4/8 ND	0.5/4 S	1 I	0.5 S	24.8 S	S	S
Ab4249	24	OXA-24, OXA-51	>32 ND	>8/4 ND	>8/4 ND	>64/4 ND	>16 R	>8/8 ND	>8/4 R	>4 R	0.5 S	18.7 R	18 S	6 R
Ec1	ND	CTX-M	0.5 S	0.25 S	0.38 S	<4 S	<0.25 S	0.064 S	0.25 S	<0.06 S	0.5 S	27 S	<1 S	<20 S
Ec6	ND	CTX-M	>64 R	0.38 S	1 S	16 R	<0.25 S	0.047 S	0.19 S	>4 R	1 S	17 R	4 S	>320 R
Ec13	ND	CTX-M	0.5 S	0.125 S	0.38 S	<4 S	<0.25 S	0.047 S	0.19 S	<0.06 S	1 S	26 S	4 S	>320 R
Kp3	ND	KPC, CTX-M	>32 R	2/4 S	>8/4 R	>64/4 R	>16 R	0.023 S	0.25/4 S	4 R	0.5 S	22.1 S	14 R	6 R
Kp6	ND	IMP, CTX-M	>32 R	>8/4 R	>8/4 R	>64/4 R	8 R	8/8 R	0.5/4 S	2 R	0.125 S	25.5 S	20 S	6 R
Kp16	ND	DHA	>32 R	0.5/2 S	2/4 S	>64/4 R	0.25-0.5 S	0.05/8 S	2/4 S	0.5 I	0.5 S	23.2 S	26 S	30 S
Pa3	235	VIM-2	>32 R	>8/4 R	>8/4 R	>64/4 R	>16 R	>8/8 R	>8/4 R	>4 R	2 S	24.6 S	11 R	6 R
Pa46	111	VIM-2	>32 R	>8/4 R	>8/4 R	>64/4 R	>16 R	>8/8 R	>8/4 R	>4 R	1 S	23.3 S	12 R	6 R
Pa1016	175		32 R	2/4 S	2/4 S	>64/4 R	16 R	>8/8 R	4S	>4 R	4 S	25.6 S	24 S	6 R

a ST, sequence type; CTZ, ceftazidime; CTV, ceftazidime-avibactam; CTT, ceftolozane-tazobactam; PIT, piperacillin-tazobactam; MER, meropenem; MEV, meropenem-vaborbactam; IMR, imipenem-relebactam; CIP, ciprofloxacin; COL, colistin; FDC, cefiderocol; AMI, amikacin; TRS, trimethoprim-sulfamethoxazole; ND, not determined; R, resistant; S, susceptible.

For biofilm susceptibility studies on silicone disks, a clinical strain of P. aeruginosa (Pa1016) was tested.

All strains were stored in skim milk at −80°C in cryovial storage containers. Prior to each experiment, the strains were subcultured in Trypticase soy agar (TSA; bioMérieux SA, Marcy l’Etoile, France) and incubated at 37°C for 24 h.

All studied strains were isolated from patients at the Vall d’Hebron University Hospital (VHUH).

### Antimicrobial agents.

**(i) AgNPs.** For the synthesis of AgNPs, the protocol described previously by Bastús et al. (34) was used, with some modifications. Briefly, 100 mL of a solution of 5 mM sodium citrate (SC; Sigma-Aldrich, Steinheim, Germany) and 1 mL of 2.5 mM tannic acid (TA; Sigma-Aldrich, Steinheim, Germany) was prepared in a three-neck round-bottom flask. The solution was heated to its boiling point under reflux using a heating mantle, with vigorous stirring. A condenser was used to prevent the evaporation of the solvent. After 20 min of boiling, 1 mL of 125 mM silver nitrate (AgNO_3_; Sigma-Aldrich, Steinheim, Germany) was added, and a color change of the solution to bright yellow was immediately observed. After 15 min, 1 mL of the solution was collected for the characterization of the sample (G00). Subsequently, 80 mL of the solution was extracted, and the remaining 20 mL was diluted with 80 mL of 5 mM SC.

To grow AgNPs, the solution was cooled to 90°C. Thereafter, five injection cycles with 0.1 mL of SC (25 mM) followed by 0.25 mL of TA (2.5 mM) and, finally, 0.20 mL of AgNO_3_ (25 mM) were performed. One minute was allowed between each addition. After 15 min of each injection cycle for the three reagents, 1 mL of the solution was collected for characterization (G01, G02, G03, G04, and G05).

**(ii) Conjugation of AgNPs with mPEG-COOH and AK.** For the conjugation of AgNPs with mPEG-COOH (Sigma-Aldrich, Steinheim, Germany), AgNPs (G05) were first purified by centrifugation at 17,000 × *g* for 15 min at 23°C and then resuspended in MilliQ water. An aqueous solution of 0.3 μM mPEG-COOH was then added to the AgNP solution and vigorously stirred overnight at 23°C. Next, AgNPs_mPEG was washed by centrifugation at 17,000 × *g* for 15 min at 23°C and resuspended in MilliQ water. Subsequently, an aqueous solution of 100 mg/L AK (Sigma-Aldrich, Steinheim, Germany) was added to AgNPs_mPEG and vigorously stirred overnight at 23°C. Finally, AgNPs_mPEG_AK was washed by centrifugation at 17,000 × *g* for 15 min at 23°C and resuspended in MilliQ water. One milliliter of each solution (AgNPs_mPEG and AgNPs_mPEG_AK) was collected for characterization.

**(iii) Characterization of AgNPs, AgNPs_mPEG, and AgNPs_mPEG_AK.** AgNPs (G00, G01, G02, G03, G04, and G05), AgNPs_mPEG, and AgNPs_mPEG_AK were fully characterized with an optimized suite of spectroscopy and microscopy methods: UV-visible spectra (UV-Vis) (Agilent Cary 60 UV-Vis spectrophotometer; Agilent Technologies), zeta potential (ZP) (ZetaSizer Nano ZS; Malvern, United Kingdom), dynamic light scattering (DLS) (ZetaSizer Nano ZS; Malvern, United Kingdom), nanoparticle tracking analysis (NTA) (NanoSight; Malvern Instruments Ltd., United Kingdom) (488-nm laser), and transmission electron microscopy (TEM) (20-keV, Scanning transmission electron microscope, Field electron and Ion Magellan 400 L Extreme resolution scanning electron microscope).

The UV-Vis spectrum was studied in the range of 300 to 800 nm, ZP was used to analyze the surface charge, and DLS and NTA were used to measure NP sizes.

TEM was used to determine the size and shape of AgNPs (G00, G01, G02, G03, G04, and G05). TEM grids were prepared as follows: 10 μL of AgNPs were drop-cast onto a copper grid and air dried. The sizes of at least 100 particles were measured using ImageJ software, and the average size and standard deviation (SD) were calculated.

**(iv) Quantification of AK in AgNPs_mPEG_AK samples.** The AK concentration in the AgNPs_mPEG_AK samples was quantified using the quantitative microsphere system (QMS) amikacin assay (Thermo Fisher Scientific, Hennigsdorf, Germany). Briefly, the assay is based on competition between the antibiotic in the sample and a microparticle coated with the antibiotic to bind to the antiamikacin antibodies present in the reagent. The rate of the absorbance change in the presence of AK was measured photometrically, and the concentration of AK was determined from a concentration-dependent classic agglutination inhibition curve.

### Planktonic susceptibility studies.

**(i) Efficacy of AgNPs_mPEG_AK determined by microdilution technique.** The MICs of the different treatments (AgNPs_mPEG, AgNPs_mPEG_AK, AgNPs_mPEG+AK, and AK alone) against A. baumannii (Ab4, Ab60, and Ab4249), E. coli (Ec1, Ec6, and Ec13), K. pneumoniae (Kp3, Kp6, and Kp16), and P. aeruginosa (Pa3, Pa46, and Pa1016) strains were determined by the broth microdilution technique according to ISO guidelines and EUCAST breakpoints (52, 53). A. baumannii ATCC 19606, K. pneumoniae ATCC 25922, and P. aeruginosa ATCC 27853 were used as quality control strains.

The concentration of Ag^+^ in AgNPs was theoretically calculated considering the concentration of Ag^+^ added in the synthesis procedure and the Ag^+^ concentration lost in the conjugation process due to the centrifugation steps.

Briefly, 50 μL of Mueller-Hinton broth (MHB) medium (Becton, Dickinson and Company, Le Pont de Claix, France) was added to each well of a 96-well microtiter plate (Deltalab SL, Barcelona, Spain), except for the first column, in which 100 μL of the corresponding treatment (AgNPs_mPEG with a final concentration of 12.5 mg/L of Ag^+^, AgNPs_mPEG_AK with final concentrations of 0.5 mg/L of AK and 12.5 mg/L of Ag^+^, AgNPs_mPEG with a final concentration of 12.5 mg/L of Ag^+^ plus AK, and AK alone) was added. The treatments were then serially diluted, and 50 μL of the inoculum was added to each well at a final concentration of 5 × 10^5^ CFU/mL. A growth control, containing only medium and the inoculum, and a sterile control, containing only medium, were prepared as quality controls. Finally, the microtiter plate was incubated for 24 h at 37°C, and the results were expressed as milligrams per liter. The experiments were performed in triplicate.

**(ii) Efficacy of AgNPs_mPEG, AgNPs_mPEG_AK, AgNPs_mPEG plus AK, and AK alone over time.** The effects of AgNPs_mPEG, AgNPs_mPEG_AK, AgNPs_mPEG+AK, and AK alone over time on two strains each of A. baumannii (Ab4 and Ab60), E. coli (Ec6 and Ec13), K. pneumoniae (Kp3 and Kp6), and P. aeruginosa (Pa46 and Pa1016) were assessed.

The protocol described previously by Pillai et al. (54) was followed, with some modifications. Briefly, sterile test tubes were prepared with MHB and the treatments at different concentrations (AgNPs_mPEG, AgNPs_mPEG_AK, AgNPs_mPEG+AK, and AK alone), and 0.25 mL of the bacterial inoculum was added to obtain a final concentration of 5 × 10^5^ CFU/mL. Growth and culture medium sterility control tubes were also prepared. All of the tubes were incubated at 37°C with vigorous shaking at 60 rpm, and the number of CFU per milliliter was determined 0, 4, 8, 24, and 48 h after the addition of the treatment by plating onto TSA. The treatment concentrations were varied by increasing the concentration when low susceptibility was observed.

The results were expressed as log_10_ CFU per milliliter. A decrease of ≥3 log_10_ CFU/mL in comparison to the initial inoculum indicated that the treatment had a bactericidal effect. If the decrease was <3 log_10_ CFU/mL, the treatment was considered bacteriostatic. Moreover, a synergistic effect was observed when there was a decrease of ≥2 log_10_ CFU/mL compared to the most effective treatment.

**(iii) Efficacy of AgNPs_mPEG_AK and hyperthermia.** The efficacy of AgNPs_mPEG (at a final concentration of 18.75 mg/L of Ag^+^), AgNPs_mPEG_AK (at final concentrations of 0.75 mg/L of AK and 18.75 mg/L of Ag^+^), and AK alone (at a final concentration of 0.75 mg/L of AK) combined with hyperthermia was assessed against one strain each of A. baumannii (Ab60), E. coli (Ec6), K. pneumoniae (Kp6), and P. aeruginosa (Pa1016).

Two 12-well plates were prepared with 3 mL/well of the treatments (AgNPs_mPEG, AgNPs_mPEG_AK, and AK alone), and 1 mL of the inoculum of the studied strains at a final concentration of 5 × 10^5^ CFU/mL was added. Both plates were tempered for 30 min at 37°C. Next, one plate was maintained at 37°C, whereas the other was treated with 1, 2, and 3 pulses at 41°C to 42°C for 15 min for each pulse, and the plate was allowed to stand for 30 min at 37°C between pulses. A thermometer (Omega Instruments, Manchester, United Kingdom) and a thermocouple probe (Sanara, Barcelona, Spain) were used to verify the temperature of the control well during the experiment. To determine the same sample collection time under both conditions, we also use the word “pulse” for the nonhyperthermia conditions, although no pulses were applied and the temperature was maintained at 37°C.

The efficacy of the treatments was determined 30 min after the application of the pulse and after 24 h by collecting a sample of 100 μL and plating it onto TSA for quantitative culture. The results are expressed as log_10_ CFU per milliliter.

### Biofilm susceptibility studies.

**(i) Biofilm formation on silicone disks.** For biofilm formation, the protocol described previously by Chandra et al. (55) was followed, with some modifications, and silicone disks (15-mm diameter by 1.5-mm thickness; Merfasa SL, Barcelona, Spain) were used as the substrates for biofilm formation. First, the biofilm-producing strain (Pa1016) was grown overnight in tryptic soy broth (TSB; Becton, Dickinson and Company, Le Pont de Claix, France) at 37°C at 60 rpm. After centrifugation at 3,500 rpm for 5 min at 4°C, the cell suspension was washed three times with sterile phosphate-buffered saline (PBS) (pH 7.2) (Merck, Darmstadt, Germany), and an inoculum of 1 × 10^7^ CFU/mL was prepared in PBS (pH 7.2). Next, 4 mL of the inoculum and silicone disks were added to each well of a 12-well plate. The plates were incubated for 90 min at 37°C (the adhesion step). The silicone disks were then placed into a new 12-well plate containing 4 mL of TSB. Finally, the plate was incubated overnight at 37°C with shaking at 60 rpm (the growth step).

**(ii) Efficacy of AgNPs_mPEG_AK and hyperthermia determined by CLSM.** The efficacy of AgNPs_mPEG, AgNPs_mPEG_AK, and AK alone combined with hyperthermia was assessed against one strain of P. aeruginosa (Pa1016) growing on the surface of the silicone disks.

After biofilm formation, two 12-well plates were prepared with 3 mL of the treatments (AgNPs_mPEG with a final concentration of 75 mg/L of Ag^+^, AgNPs_mPEG_AK with final concentrations of 3 mg/L of AK and 75 mg/L of Ag^+^, and AK at a final concentration of 3 mg/L) and 1 mL of TSB, and silicone disks with biofilms formed were added to each well. For this experiment, AgNPs_mPEG and AgNPs_mPEG_AK were concentrated 4-fold (centrifuged at 17,000 × *g* for 15 min) to achieve significant efficacy. Both plates were then tempered for 30 min at 37°C before hyperthermia was applied. One plate was maintained at 37°C, whereas the other was treated with 3 pulses at 41°C to 42°C for 15 min, leaving the plate for 30 min at 37°C between pulses. A thermometer and a thermocouple probe were used to verify the temperature of the control disk during the experiment.

The effect of the treatment was visualized 24 h after the last pulse using a Zeiss LSM980 confocal laser scanning microscope with excitation wavelengths of 488 nm and 568 nm. Biofilms were stained using a Live/Dead BacLight viability kit (Molecular Probes, Invitrogen, Leiden, The Netherlands), according to the manufacturer’s instructions, which consisted of staining the samples with 200 μL of a mixture of SYTO 9 (3.34 mM solution in dimethyl sulfoxide [DMSO]) and propidium iodide (20 mM solution in dimethyl sulfoxide) and incubating them at 23°C in the dark for 30 min. The mixture was prepared using 3 μL of each dye per 1 mL of sterile distilled water. Three areas of the biofilm on each silicone disk were randomly chosen and scanned with a 2-μm step size in order to take representative images of the sample and different areas of the disks. Simultaneous dual-channel imaging was used to display green (live cells) and red (dead cells) fluorescence. ZEN microscope software from Zeiss (LSM980) was used to create a z-projection view of the formed biofilms, and the ImageJ (1.45s) software package was used to calculate the values for live (green) and dead (red) cells. All experiments were performed in triplicate to ensure the reliability of the results. When possible, the microscope operator and the person who prepared the samples were not the same.

The results are expressed as percent cell viability and were analyzed using one-way analysis of variance (ANOVA) and Tukey’s *post hoc* test. Statistical analyses were performed using the Statistical Package for the Social Sciences (SPSS; SPSS Inc., Chicago, IL, USA). Statistical significance was set at a *P* value of ≤0.05.
